# Синдром гипокальциурической гиперкальциемии. Редкость ли? Два клинических случая в амбулаторной практике

**DOI:** 10.14341/probl13125

**Published:** 2022-06-17

**Authors:** М. А. Свиридонова

**Affiliations:** Общество с ограниченной ответственностью «Огни Олимпа»

**Keywords:** семейная гипокальциурическая гиперкальциемия, кальций-чувствительный рецептор, CASR, первичный гиперпаратиреоз

## Abstract

Синдром гипокальциурической гиперкальциемии (Familial hypocalciuric hypercalcemia, FHH) является наследственным заболеванием, в основе которого лежит дисфункция кальций-чувствительного рецептора или ассоциированных с ним белковых комплексов. По последним данным, распространенность этого состояния может быть сопоставимой с частотой первичного гиперпаратиреоза. Клинические проявления FHH, как правило, отсутствуют, но в ряде случаев могут отмечаться классические симптомы гиперкальциемии. Своевременная дифференциальная диагностика FHH позволяет избежать дорогостоящего инструментального обследования, а также необоснованного хирургического лечения. Представленные в данной публикации клинические случаи демонстрируют неоправданные сложности в этом вопросе и необходимость повышения информированности врачей о синдроме семейной гипокальциурической гиперкальциемии.

## Abstract (en)

Hypocalciuric hypercalcemia syndrome (familial hypocalciuric hypercalcemia, FHH) is an inherited condition based on dysfunction of the calcium receptor or its associated partner proteins. Recent evidence suggests that the prevalence of this condition may be comparable to that of primary hyperparathyroidism. Clinical manifestations of FHH are usually absent; however the classic symptoms of hypercalcemia may be present in some cases. Timely differential diagnosis of FHH avoids unnecessary and expensive instrumental examination, as well as ineffective treatment. The clinical cases presented in this publication demonstrate the unjustified difficulties in this issue and the necessity to raise the awareness of physicians about the familial hypocalciuric hypercalcemia.

## АКТУАЛЬНОСТЬ

В настоящее время гиперкальциемия является распространенной лабораторной находкой, частота которой среди взрослого населения составляет около 3% [1]. В структуре причин гиперкальциемии на первом месте располагается первичный гиперпаратиреоз (ПГПТ). По эпидемиологическим данным, распространенность ПГПТ находится в пределах 34–120 случаев на 100 000 женщин и 13–36 случаев на 100 000 мужчин [2].

На втором месте среди причин повышения кальция крови предположительно находятся онкологические заболевания. Паранеопластическая гиперкальциемия может быть обусловлена как метастатическим поражением скелета, так и костной резорбцией под влиянием ПТГ-подобного пептида, который продуцируют некоторые опухоли. Кроме того, при ряде гемобластозов (миеломной болезни, лимфомах, лимфогранулематозе, лейкозах) усиливается образование активных форм витамина D из-за присутствия в опухолевых клетках 1-альфа-гидроксилазы. Высокие концентрации активных метаболитов витамина D также стимулируют резорбцию костной ткани и всасывание кальция в кишечнике, приводя к гиперкальциемии [1].

Одной из редких причин повышения кальция в крови считается синдром семейной гипокальциурической гиперкальциемии (Familial hypocalciuric hypercalcemia, FHH). Впервые этот синдром был описан в 1972 г. и считался наследуемым по аутосомно-доминантному механизму [3][4]. Однако в последнее время появились данные и о рецессивном типе наследования [5].

В основе развития FHH лежит генетический дефект, приводящий к нарушению функции кальций-чувствительного рецептора (CаSR), в результате которого растормаживается секреция паратгормона (ПТГ), повышается уровень кальция крови, но не происходит усиления экскреции кальция с мочой [6].

В большинстве случаев FHH протекает бессимптомно и сопровождается невыраженными клиническими проявлениями. Но своевременная дифференциальная диагностика этого состояния является краеугольным камнем в определении дальнейшей лечебной тактики. В отличие от ПГПТ, хирургическое лечение при FHH не купирует гиперкальциемию и лишено какого-либо смысла [7][8].

Из-за преимущественно субклинического характера нарушений, возникающих при FHH, говорить о его реальной распространенности до настоящего времени достаточно сложно [9].

Однако, согласно недавнему исследованию, частота выявления мутаций в гене CaSR, соответствующих синдрому FHH, в когорте из 51 289 лиц составила 74,1 на 100 000 [9]. Эта цифра вполне сопоставима со скорректированной по возрасту распространенностью ПГПТ (48 на 100 000 для мужчин и 120 на 100 000 для женщин) [10].

## РЕГУЛЯЦИЯ ОБМЕНА КАЛЬЦИЯ В ОРГАНИЗМЕ

Внеклеточный кальций является универсальным регулятором многих жизненно важных процессов, в том числе нервно-мышечной передачи, свертывания крови и минерализации костной ткани. Внутриклеточный кальций в первую очередь необходим для работы мышечной ткани и секреции гормонов [11]. Для бесперебойного обеспечения этих процессов кальцием в организме сформирована система поддержания его постоянных концентраций как во внутриклеточном, так и внеклеточном пространствах [12].

Эта система состоит из пяти основных компонентов (рис. 1):

1 — паращитовидные железы, являющиеся детекторами уровня кальция в крови и источниками ПТГ;

2 — ПТГ и 1,25-дигидроксивитамин D3 (кальцитриол), которые опосредуют взаимодействия между паращитовидными железами, костной тканью, почками и кишечником;

3 — костная ткань, которая представляет собой основной резервуар кальция в организме и является буфером для краткосрочных изменений его концентраций;

4 — почки, которые обеспечивают перемещение кальция между внешней средой и внеклеточной жидкостью, а также являются местом образования активной формы витамина D (кальцитриола);

5 — кишечник, который обеспечивает поступление кальция в организм из пищи.

**Figure:**
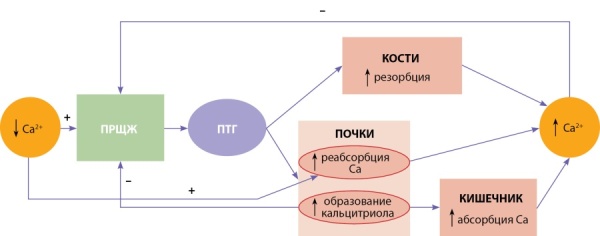
Рисунок 1. Гомеостаз кальция в организме.ПРЩЖ — паращитовидные железы; ПТГ — паратгормон.Figure 1. Calcium homeostasis in the body.

При снижении уровня кальция в сыворотке крови из паращитовидных желез высвобождается ПТГ, приводящий к следующим изменениям:

Кроме того, низкие концентрации кальция в сыворотке крови стимулируют его реабсорбцию в почках, независимо от эффектов ПТГ [13][14].

Все вышеперечисленные процессы приводят к повышению кальция в крови и по механизму отрицательной обратной связи, наряду с кальцитриолом, подавляют секрецию ПТГ [12].

## СТРОЕНИЕ И ОРГАНИЗАЦИЯ РАБОТЫ КАЛЬЦИЙ-ЧУВСТВИТЕЛЬНОГО РЕЦЕПТОРА

Кальций-чувствительный рецептор (CaSR) человека представляет собой димерный белок, расположенный на клеточной мембране. Он относится к суперсемейству G-протеин-связанных рецепторов и состоит из 1078 аминокислот [15]. Максимальная экспрессия CaSR отмечается в паращитовидных железах и почках [16].

Рецептор содержит большой внеклеточный домен (612 аминокислот), ответственный за связывание лиганда и димеризацию, трансмембранный домен, состоящий из семи спиралей, и меньший внутриклеточный домен (216 аминокислот), который передает сигнал «нижестоящим» внутриклеточным белкам-партнерам [17].

Присоединение лиганда к внеклеточной части рецептора инициирует конформационные изменения, приводящие к активации одного из G-белков, связанного с внутриклеточным доменом рецептора. Переход G-белка в активное состояние запускает каскад внутриклеточных реакций. Главным внутриклеточным эффектором передачи этого сигнала является цитоплазматический ионизированный кальций [18].

На рисунке 2 схематично представлены строение и функционирование CaSR. Активация α11-субъединицы G-белка стимулирует фосфолипазу C-β (PLCβ), которая катализирует образование инозитол-1,4,5-трифосфата (IP3) и диацилглицерола (DAG)из фосфатидилинозитол-4,5-бисфосфата (PIP2). Накопление IP3 опосредует быстрое высвобождение кальция в цитозоль из внутриклеточных хранилищ, тогда как DAG активирует каскад MAPK (митоген-активируемой протеин-киназы). Эти внутриклеточные процессы приводят к снижению секреции ПТГ в клетках паращитовидных желез и снижению реабсорбции кальция в канальцах почек [19].

**Figure:**
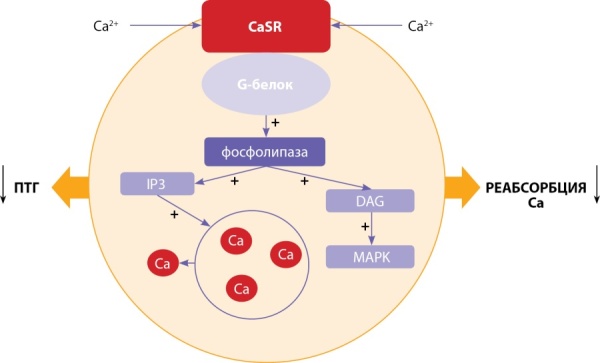
Рисунок 2. Функционирование кальций-чувствительного рецептора.Ca — кальций; CaSR — кальций-чувствительный рецептор; IP3 — инозитол-1,4,5-фосфат; DAG — диацилглицерол; MAPK — митоген-активируемая протеинкиназа; ПТГ — паратгормон.Figure 2. Functioning of the calcium-sensing receptor.

Достаточная экспрессия CaSR на поверхности клеток является необходимым условием для его нормального функционирования. Уровень экспрессии рецепторов на клеточной мембране зависит от интенсивности их стимуляции всеми агонистами (кальцием, магнием, L-аминокислотами, полиаминами, глутамилпептидами, фосфат- и сульфат-ионами) [19][20], а также активности эндоцитарного комплекса, включающего клатрин, β-аррестин и комплекс AP2, которые отвечают за эндосомно-лизосомную деградацию или повторное представление CaSR на поверхности клетки [21].

Мутации в генах, кодирующих CaSR и белки-партнеры, могут приводить как к снижению порога чувствительности к внеклеточному кальцию, так и его повышению. При снижении чувствительности к кальцию развивается синдром гипокальциурической гиперкальциемии, а при повышении — синдром гипокальциемии, порой сопровождающийся гиперкальциурией [10].

Синдром семейной гипокальциурической гиперкальциемии (FHH) — это генетически детерминированная группа нарушений чувствительности к внеклеточному кальцию, которая характеризуется легкой, как правило, бессимптомной гиперкальциемией и относительной гипокальциурией [22]. В настоящее время известны три типа FHH (FHH1, FHH2, FHH3). Их относительная распространенность оценивается как 64:1:10 соответственно [23].

## Семейная гипокальциурическая гиперкальциемия 1 типа

Причиной FHH1 является герминальная мутация гена CaSR, расположенного на хромосоме 3q21.1.

Это наиболее распространенная форма FHH, лежащая в основе 85% случаев этого синдрома. К настоящему времени известно более 300 мутаций гена CaSR, большинство из которых представлено точечными миссенс-мутациями в первых 350 нуклеотидах, кодирующих внеклеточный домен рецептора [24].

Результаты исследований свидетельствуют о том, что 50% мутаций в гене CaSR сопровождаются нарушениями рецепторной передачи сигналов и изменениями уровня экспрессии рецепторов на клеточной мембране [24].

Клинически FHH1 характеризуется легкой непрогрессирующей гиперкальциемией, нормальным (в 80% случаев) или немного повышенным (в 20% случаев) уровнем ПТГ в крови, а также сниженной почечной экскрецией кальция [25].

У большинства пациентов с FHH1 симптомы отсутствуют, хотя в зависимости от степени повышения кальция в крови могут наблюдаться полидипсия, полиурия, хроническая усталость, диагностироваться хронический панкреатит, желчно- и мочекаменная болезнь, хондрокальциноз. Гиперкальциемия при выраженной симптоматике может требовать коррекции. В ряде таких случаев описана эффективность терапии кальцимиметиками [26][27].

Паратиреоидэктомия при FHH1, в отличие от таковой при ПГПТ, не приводит к снижению уровня кальция в крови и не должна выполняться взрослым пациентам.

Для проведения грамотной дифференциальной диагностики FHH достаточно лабораторных методов обследования. На первый план в этом вопросе выходит исследование суточной экскреции кальция с мочой. Поскольку сама по себе экскреция кальция за определенный интервал времени в значительной степени зависит от скорости клубочковой фильтрации и продолжительности сбора мочи, общая экскреция кальция не является универсальным показателем для дифференциальной диагностики.

Для этой цели используется расчет отношения почечного клиренса кальция к клиренсу креатинина (UCCR), который при FHH составляет менее 0,01.

Для расчета используют следующую формулу:

CaCl/CrCl = [Cau x Crs]/[ Cas x Cru],

где CaCl — клиренс кальция; CrCl — клиренс креатинина; Cau — концентрация кальция в моче (ммоль/л); Crs — концентрация креатинина в сыворотке крови (мкмоль/л); Cas — концентрация кальция в сыворотке крови (ммоль/л); Cru — концентрация креатинина в моче (мкмоль/л) [28][29].

При ПГПТ индекс UCCR обычно составляет более 0,02, а значения в диапазоне 0,01–0,02 считаются «серой зоной». По результатам исследования S.E. Christensen и соавт. значения UCCR в этом интервале наблюдаются у 33% пациентов с ПГПТ и у35% с FHH [30]. В таких ситуациях для окончательной постановки диагноза требуется генетическое исследование [31].

Проявлением крайней степени снижения функции CaSR является тяжелый неонатальный гиперпаратиреоз (neonatal severe hyperparathyroidism, NSHPT). При этом редком состоянии гиперпаратиреоз начинает развиваться уже внутриутробно, так как нормальный уровень кальция в материнской крови воспринимается как низкий. В течение нескольких дней или недель после родов концентрация кальция в крови новорожденного резко возрастает, и появляется выраженная клиническая симптоматика: гипотония, вялое сосание, нарушение глотания, развитие респираторного дистресс-синдрома. Очень высокая концентрация ПТГ при NSHPT может приводить к жизнеугрожающему повышению кальция, требующему тотальной паратиреоидэктомии [32][33].

## Семейная гипокальциурическая гиперкальциемия 2 типа

Как известно, передача сигнала от кальциевого рецептора опосредуется G-белком. Мутации в генах, кодирующих различные субъединицы G-белка, также могут приводить к FHH.

FHH2 — редкое аутосомно-доминантное расстройство, вызываемое мутациями гена GNA11 (19p13), кодирующего α11-субъединицу G-белка. На сегодняшний день известно четыре различных мутации этого гена (Thr54Met, Leu135Gln, Phe220Ser, Ile200del) [34–36].

По данным литературы, у пациентов с FHH2 определяется легкая бессимптомная гиперкальциемия (<2,80 ммоль/л) [34][35]. При этом в ходе экспериментального исследования было показано, что у пациентов с мутацией Phe220Ser применение кальцимиметиков эффективно корректирует нарушенную передачу сигналов от кальция через клеточную мембрану и нормализует уровень кальция и паратгормона в крови [36].

## Семейная гипокальциурическая гиперкальциемия 3 типа

Причиной FHH3 является герминальная мутация в гене AP2S1 (19q13.3), кодирующем 2σ-субъединицу адапторного белкового комплекса, играющего ключевую роль в клатрин-зависимом эндоцитозе кальций-чувствительного рецептора. Гетерозиготные мутации в AP2S1 выявляются у 13–20% пациентов с FHH при отсутствии мутаций в CаSR [6][37][38].

Интересно, что дефектная 2σ-субъединица приводит к нарушению передачи сигналов от кальция внутрь клетки, несмотря на повышенную экспрессию CaSR на клеточной мембране [19][37]. Объяснить этот феномен позволили результаты недавнего исследования, подтвердившие гипотезу о том, что работа кальциевого рецептора обусловлена не только немедленным ответом со стороны плазматической мембраны, но и поддерживающими сигналами от эндосом. Такое явление характерно и для других рецепторов, сопряженных с G-белком [39].

Клинически пациенты с FHH3 обычно имеют более тяжелую гиперкальциемию, гипермагниемию и более выраженную гипокальциурию, чем пациенты с FHH1. Известны 3 миссенс-мутации, приводящие к FHH3, — Arg15Cys, Arg15His и Arg15Leu, среди которых Arg15Leu ассоциирована с наиболее высоким уровнем кальция в сыворотке крови [40].

При FHН3 также были описаны снижение минеральной плотности костной ткани и нарушения когнитивных функций [40].

Кроме того, среди причин гипокальциурической гиперкальциемии рассматривают образование блокирующих антител к CaSR. Вероятность такого патогенетического варианта FHH не следует сбрасывать со счетов при отягощенном семейном анамнезе по аутоиммунным заболеваниям [41].

## КЛИНИЧЕСКИЕ СЛУЧАИ

## Клинический случай №1

Пациент В., 45 лет, обратился к эндокринологу по направлению врача общей практики. Из анамнеза известно, что около 6 лет назад впервые выявлены пограничное повышение уровня кальция в крови (до 2,58 ммоль/л) и повышение сывороточной концентрации паратгормона (до 11,5 пмоль/л).

При дальнейшем обследовании обнаружен дефицит витамина D, что послужило поводом расценить состояние как вторичный гиперпаратиреоз.

Однако через полгода, после нормализации уровня витамина D, концентрация паратгормона осталась повышенной (12,5 пмоль/л), а уровень общего кальция составил 2,63 ммоль/л.

В связи с этим пациент был направлен на сцинтиграфию паращитовидных желез, при которой был обнаружен очаг накопления радиофармпрепарата позади левой доли щитовидной железы.

В дальнейшем пациент проконсультирован хирургом, который рекомендовал оперативное лечение. Однако в связи с обострением болезни Крона операция была отложена на неопределенное время.

К вопросу удалось вернуться только через 5 лет. Было рекомендовано повторное обследование, результаты которого представлены в таблице 1.

**Table:** Таблица 1. Результаты обследования пациента В. в динамикеTable 1. The results of the examination of patient V. in dynamics

Параметр	Результат(при первичном обращении)	Результат(при обращении через год)	Референсные значения
Анализы крови
Общий Ca, ммоль/л	2,59	2,61	2,15–2,50
Ca2+, ммоль/л	1,39	1,32	1,18–1,32
Фосфор, ммоль/л	0,96	1,05	0,78–1,42
Магний, ммоль/л	0,9	-	0,66–1,07
Альбумин, г/л	40,6	40,2	35–52
Креатинин, мкмоль/л	99,2	100,6	62–115
СКФ, мл/мин/1,73 м2	79	74	-
Щелочная фосфатаза, Ед/л	88	90	53–128
Остеокальцин, нг/мл	31,25	-	14–42
Дезоксипиридинолин, нмоль, ДПИД/ммоль креатинина	4,33	-	1,8–11,9
Паратгормон, пмоль/л	11	13,3	1,7–6,4
Витамин D, нг/мл	30,9	44.7	30–100
ТТГ, мЕд/л	2,4	-	0,4–4
Анализы суточной мочи
Кальций, ммоль/сут	2,46	2,01	2,5–7,5
Кальций, ммоль/л	1,64	1,25	-
Креатинин, ммоль/сут	16,27	17,46	7,1–17,7
Креатинин, ммоль/л	10,85	10,9	-

При выполнении рентгеновской денситометрии данных за остеопороз получено не было: минеральная плотность костной ткани в шейке бедренной кости (Neck) -1,1 SD, в поясничном отделе позвоночника (L1–L4) -1,8 SD, в дистальном отделе лучевой кости (Radius 33%) -0,2 SD по Z-критерию.

Расчет соотношения клиренса кальция к клиренсу креатинина позволил диагностировать семейную гипокальциурическую гиперкальциемию. При первичном обращении это соотношение составило 0,006, при обращении через год — 0,004.

Пациенту было рекомендовано генетическое обследование. Однако при секвенировании гена CaSR мутаций обнаружить не удалось, а исследование генов GNA11 и AP2S1 в России пока недоступно.

В то же время факт выявления умеренной гиперкальциемии у двух ближайших родственников хоть и не подтверждает, но свидетельствует в пользу генетического характера заболевания у данного пациента.

## Клинический случай №2

Пациентка С., 25 лет, обратилась к врачу в связи с необъяснимой прибавкой в весе. При обследовании были выявлены гипотиреоз (уровень тиреотропного гормона (ТТГ) — 11 мЕд/л), повышение количества антител к тиреопероксидазе и уровня кальция в сыворотке крови (общ. Ca — до 2,72 ммоль/л, ионизированного Cа — до 1,49 ммоль/л).

Через 3 мес терапии левотироксином уровень ТТГ нормализовался (1,02 мЕ/л), а концентрация общего кальция составила 2,61 ммоль/л на фоне нормальной продукции паратгормона (5,8 пмоль/л).

В связи со стойкой гиперкальциемией, которую не удалось объяснить нарушениями в работе паращитовидных желез, пациентке было рекомендовано обследование для исключения онкологических заболеваний.

При ультразвуковом исследовании щитовидной и паращитовидных желез, магнитно-резонансной томографии брюшной полости и малого таза, а также компьютерной томографии грудной клетки удалось выявить лишь признаки аутоиммунного поражения щитовидной железы и небольшой конкремент правой почки (2–3 мм).

Спустя 4 мес после первичного выявления гиперкальциемии пациентка обратилась в нашу клинику для получения второго мнения. Было выполнено обследование, результаты которого представлены в таблице 2.

**Table:** Таблица 2. Результаты обследования пациентки С.Table 2. Results of the examination of patient C.

Параметр	Результат	Референсные значения
Анализы крови
Общий Ca, ммоль/л	2,58	2,15–2,50
Ca2+, ммоль/л	1,33	1,18–1,32
Фосфор, ммоль/л	1,06	0,78–1,42
Магний, ммоль/л	0,87	0,66–1,07
Альбумин, г/л	40	35–52
Креатинин, мкмоль/л	65	62–115
СКФ, мл/мин/1,73 м2	113	-
Щелочная фосфатаза, Ед/л	78	53–128
Паратгормон, пмоль/л	5,8	1,7–6,4
Витамин D, нг/мл	17,3	30–100
ТТГ, мЕд/л	0,7	0,4–4
Анализы суточной мочи
Кальций, ммоль/сут	1,19	2,5–7,5
Кальций, ммоль/л	0,7	-
Креатинин, ммоль/сут	14,28	7,1–17,7
Креатинин, ммоль/л	8,4	-

Полученная низкая концентрация кальция в суточной моче побудила пациентку самостоятельно повторить анализ, результаты которого оказались аналогичными (1,36 ммоль/сут).

Отношение клиренса кальция к клиренсу креатинина составило 0,002, что соответствует синдрому FHH.

К сожалению, возможность подтвердить диагноз генетически и обследовать родственников на предмет гиперкальциемии у пациентки отсутствовала, однако для определения дальнейшей тактики ее ведения полученной информации оказалось достаточно.

Постепенная нормализация уровня витамина D с помощью препарата колекальциферола в дальнейшем не привела к клинически значимому изменению уровня кальция крови через 3 мес (через 3 мес уровень общ. Ca составил 2,61 ммоль/л, альбумина — 41 г/л).

## ОБСУЖДЕНИЕ

Скрининговая оценка уровня кальция сыворотки крови показана широкому кругу лиц. В соответствии с современными представлениями это исследование целесообразно при жалобах на общую и мышечную слабость, судороги, боли в костях имышцах, хронической диспепсии, остеопорозе, низкотравматичных переломах, мочекаменной болезни и т.д. [1].

При выявлении гиперкальциемии следующим этапом диагностики является определение уровня ПТГ в крови [1]. Зачастую уже на основании этих исследований делаются выводы о диагнозе и определяется направление дальнейших лечебных мероприятий. Оценкой кальция в суточной моче, а тем более расчетом соотношения между клиренсом кальция и креатинина нередко пренебрегают.

В первом из представленных клиническом случае диагностический поиск осложнился наличием подозрительных очагов гиперфиксации радиофармпрепарата при сцинтиграфии паращитовидных желез. Эта находка могла бы служить подтверждением наличия у пациента ПГПТ, если бы согласно алгоритму дифференциальной диагностики гиперкальциемии была исследована суточная экскреция кальция с мочой [1].

К счастью, обострение сопутствующего заболевания уберегло пациента от бессмысленной операции, а выявление гипокальциурии в дальнейшем заставило пересмотреть диагностическую концепцию и тактику лечения пациента.

Во втором клиническом случае из-за нормального уровня ПТГ диагностика и вовсе пошла в ложном направлении. Второй по распространенности (после ПГПТ) причиной гиперкальциемии считается паранеопластический синдром [1]. Поэтому в свете отсутствия других причин гиперкальциемии в клинической картине и результатов первичного обследования (пациентка не принимала никаких препаратов, способных повлиять на уровень кальция крови, отклонений в работе почек, гипервитаминоза D, тиреотоксикоза выявлено не было) онкопоиск мог бы быть оправдан. Однако в диагностике был упущен важный этап — оценка суточной экскреции кальция с мочой. Если бы гипокальциурия и столь низкое соотношение UCCR были выявлены вовремя, эмоционально и финансово затратного обследования на предмет выявления онкологических заболеваний можно было бы избежать.

К сожалению, амбулаторное звено частной системы здравоохранения лишено возможностей проведения обследования на бесплатной для пациентов основе, поэтому привлечь к углубленному обследованию родственников первого пациента, а также выполнить генетическое исследование во втором случае не удалось. Однако тех рутинных анализов крови и мочи, которые были выполнены, оказалось достаточно для дифференциальной диагностики синдрома гиперкальциемии в обоих случаях.

## ЗАКЛЮЧЕНИЕ

Синдром семейной гипокальциурической гиперкальциемии считается одной из редких причин повышения кальция в крови. Однако в свете новых научных и клинических данных распространенность этого синдрома кажется недооцененной.

При выявлении гиперкальциемии обязательным диагностическим мероприятием наряду с определением уровня паратгормона в крови должна быть оценка суточной экскреции кальция (а при ее низких или нормальных значениях — расчет отношения клиренса кальция к клиренсу креатинина).

Недостаточная информированность специалистов в этом вопросе приводит к ненужным диагностическим мероприятиям и назначению необоснованного хирургического лечения. Молекулярно-генетическое исследование позволяет в ряде случаев уточнить диагноз, однако является дорогостоящим и не полностью охватывает возможные генетические изменения.

Представленные в статье клинические ситуации являются наглядным примером постановки диагноза на основании клинической картины и рутинных лабораторных данных.

## ДОПОЛНИТЕЛЬНАЯ ИНФОРМАЦИЯ

Источник финансирования. Работа выполнена по инициативе автора без привлечения финансирования.

Конфликт интересов. Автор одобрил финальную версию статьи перед публикацией, выразил согласие нести ответственность за все аспекты работы, подразумевающую надлежащее изучение и решение вопросов, связанных с точностью или добросовестностью любой части работы.

Согласие пациента. Пациенты добровольно подписали информированное согласие на публикацию персональной медицинской информации в обезличенной форме в журнале «Проблемы эндокринологии».
